# Assessment of the Drug Interaction Potential of Unconjugated and GalNAc_3_-Conjugated 2′-MOE-ASOs

**DOI:** 10.1016/j.omtn.2017.08.012

**Published:** 2017-08-30

**Authors:** Colby S. Shemesh, Rosie Z. Yu, Mark S. Warren, Michael Liu, Mirza Jahic, Brandon Nichols, Noah Post, Song Lin, Daniel A. Norris, Eunju Hurh, Jane Huang, Tanya Watanabe, Scott P. Henry, Yanfeng Wang

**Affiliations:** 1 Department of Pharmacokinetics and Clinical Pharmacology, Ionis Pharmaceuticals, 2855 Gazelle Ct., Carlsbad, CA 92010, USA; 2 Optivia Biotechnology, Inc., 3010 Kenneth St., Santa Clara, CA 95054, USA; 3 Alliance Pharma, 17 Lee Boulevard, Malvern, PA 19355, USA

**Keywords:** antisense oligonucleotides, pharmacokinetics, drug interaction, metabolism, transport

## Abstract

Antisense oligonucleotides are metabolized by nucleases and drug interactions with small drug molecules at either the cytochrome P450 (CYP) enzyme or transporter levels have not been observed to date. Herein, a comprehensive in vitro assessment of the drug-drug interaction (DDI) potential was carried out with four 2′-O-(2-methoxyethyl)-modified antisense oligonucleotides (2′-MOE-ASOs), including a single triantennary N-acetyl galactosamine (GalNAc_3_)-conjugated ASO. Several investigations to describe the DDI potential of a 2′-MOE-ASO conjugated to a high-affinity ligand for hepatocyte-specific asialoglycoprotein receptors are explored. The inhibition on CYP1A2, CYP2B6, CYP2C8, CYP2C9, CYP2C19, CYP2D6, CYP2E1, and CYP3A4 and induction on CYP1A2, CYP2B6, and CYP3A4 were investigated in cryopreserved hepatocytes using up to 100 μM of each ASO. No significant inhibition (half maximal inhibitory concentration [IC_50_] > 100 μM) or induction was observed based on either enzymatic phenotype or mRNA levels. In addition, transporter interaction studies were conducted with nine major transporters per recommendations from regulatory guidances and included three hepatic uptake transporters, organic cation transporter 1 (OCT1), organic anion transporting polypeptide 1B1 (OATP1B1), and OATP1B3; three renal uptake transporters, organic anion transporter 1 (OAT1), OAT3, and OCT2; and three efflux transporters, P-glycoprotein (P-gp), breast cancer resistance protein (BCRP), and bile salt export pump (BSEP). None of the four ASOs (10 μM) were substrates of any of the nine transporters, with uptake <2-fold compared to controls, and efflux ratios were below 2.0 for BCRP and P-gp. Additionally, neither of the four ASOs showed meaningful inhibition on any of the nine transporters tested, with the mean percent inhibition ranging from −38.3% to 24.2% with 100 μM ASO. Based on these findings, the unconjugated and GalNAc_3_-conjugated 2′-MOE-ASOs would have no or minimal DDI with small drug molecules via any major CYP enzyme or drug transporters at clinically relevant exposures.

## Graphical Abstract

**Figure:**
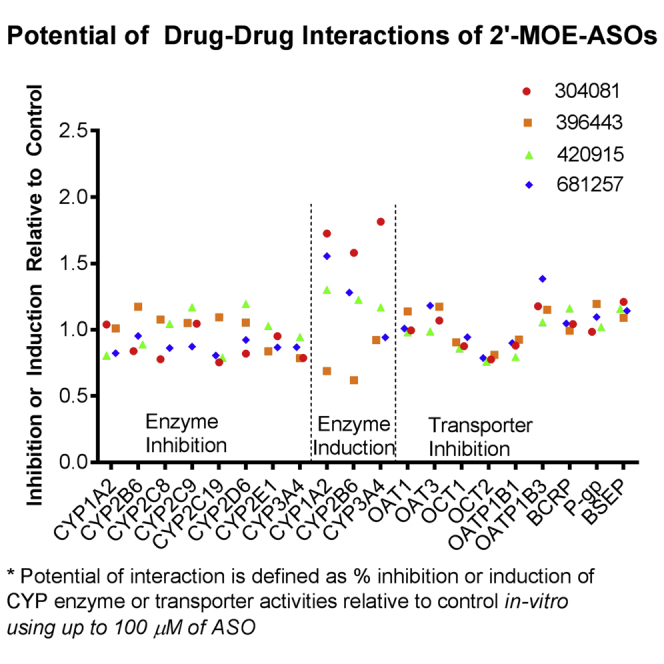

## Introduction

Antisense oligonucleotides (ASOs) are a growing class of versatile biomolecules, which have garnered much attention in the past decade as a mature and attractive platform for therapeutic drug development. Nearly 30 years of exhaustive research into antisense technology have advanced the platform into a rapid development stage for the treatment of a broad range of diseases, including severe and rare genetic disorders, cancers, cardiovascular and metabolic illnesses, and infections.^1^, ^2^, ^3^ ASOs are considered the most direct therapeutic strategy to hybridize target RNA, and, as such, to no surprise, ASOs compose the majority of investigational new drug (IND) submissions for nucleic-acid-based therapeutics.^4^ Significant advancements in ASO chemistries have fostered a wide range of modifications with an improved understanding of ASO pharmacology, pharmacokinetics (PKs), and toxicology, which collectively have led to widespread use of ASOs within broadened clinical pipelines.^5^, ^6^, ^7^ ASOs undergo Watson-Crick hybridization to bind to cognate RNA sequences, which could modulate gene expression or translation of proteins that are in question.^8^, ^9^, ^10^ ASOs function through a wide variety of mechanisms, such as the RNase H degradation pathway to achieve the desired pharmacological effect.^11^ Pivotal modifications to backbone and base pair chemistries have included the use of phosphorothioate (PS) and 2′-MOE-ASOs, which increase overall tolerance and potency. PS modifications increase nuclease resistance and extend circulating half-life, whereas 2′-MOE-ASOs have ribose sugar modifications at the 5′ and 3′ termini, which increase resistance to exonuclease cleavage and enhance binding to target mRNA. Lastly, and as one of the most compelling successes in oligonucleotide drug development, triantennary N-acetyl galactosamine (GalNAc_3_)-conjugated ASOs allow for efficient delivery, with high affinity to asialoglycoprotein receptors (ASGPRs) for liver targeting.^12^ Remarkably over 20- to 30-fold improved potency of GalNac_3_-ASO conjugates compared to unconjugated ASOs have been observed in vivo.^13^

The PK properties of PS and 2′-MOE-ASOs are widely comparable, highly predictable, extrapolatable, and well documented across species in preclinical and clinical findings.^14^, ^15^, ^16^, ^17^, ^18^ Nevertheless, because ASO therapies are largely in the development stage, only a limited number of reported drug-drug interaction (DDI) studies between unconjugated ASOs and other drugs have been reported, and no accounts for DDI studies with GalNAc_3_-conjugated ASOs exist.^19^, ^20^, ^21^, ^22^ Published findings of clinical DDI studies have investigated the potential interactions of unconjugated ASOs with co-medications that are often used in the disease populations under study. These co-medications have included simvastatin, ezetimibe, rosiglitazone, glipizide, metformin, cisplatin, and gemcitabine, which collectively utilize diverse clearance routes, including cytochrome P450 3A4 (CYP3A4), glucoronidation, CYP2C8/C9, CYP2C9/C8, renal, and nucleoside kinases. The results of these studies have shown no reported cases of known clinical interactions between unconjugated ASOs and co-medications.

Currently, there are no specific regulatory guidances on clinical pharmacology studies for nucleic-acid-based therapeutics, and the DDI panel recommendations are similar to those for small molecules, which include in vitro induction and inhibition screens for the major CYP enzymes and substrate and inhibition investigations for the major drug transporters to evaluate the need for in vivo studies.^23^, ^24^ DDIs can occur when one drug alters the uptake or metabolism of a co-administered drug, leading to altered PKs and pharmacology. Drugs that are substrates or inhibitors of these transporters or inducers or inhibitors of the major CYP enzymes may cause adverse drug reactions if co-medications, foods, or supplements are also substrates or inhibitors of the same transporters or inducers or inhibitors of CYPs.^25^

Within this research, an extensive investigation across a diverse panel of ASOs is conducted to evaluate a total of four distinctive ASOs (one recently approved and three currently in clinical development), including a GalNAc_3_-conjugated-ASO (Table 1). CYP1A2, CYP2B6, CYP2C8, CYP2C9, CYP2C19, CYP2D6, CYP2E1, and CYP3A4 inhibition potential using human primary hepatocytes and CYP1A2, CYP2B6, and CYP3A4 induction potential at both the enzyme activity level and the mRNA level were assessed. Additionally, the cellular level exposure of each respective ASO in the hepatocytes was also evaluated under the same conditions used in the inhibition experiments to ensure adequate uptake. For transporter studies, the potential for ASOs as substrates or inhibitors of major drug transporters was also examined, including organic anion transporters (OAT1 and OAT3), organic cation transporters (OCT1 and OCT2), organic anion transporting polypeptides (OATP1B1 and OATP1B3), breast cancer resistance protein (BCRP), P-glycoprotein (P-gp), and the bile salt export pump (BSEP). These in vitro CYP and cell-based transporter assays provide mechanistic insights into the lack of cytochrome-P450-related DDI as well as the lack of transporter-related drug interactions with the 2′-MOE-ASOs (both unconjugated and GalNAc_3_ conjugated), providing better confidence for the safety profiles of ASOs.

**Table 1:** Characteristics of ASOs Investigated in CYP and Transporter Studies

Study Number	Compound	Oligonucleotide Sequence	Oligomer Length	Chemistry	Molecular Weight (Da)	Target Indication
1	ISIS 304801	AGCTTCTTGTCCAGCTTTAT	20-mer	2′MOE gapmer PS backbone	7,165.21	hypertriglyceridemia
2	ISIS 396443	TCACTTTCATAATGCTGG	18-mer	Uniform MOE w/PS backbone	7,127.32	spinal muscular atrophy
3	ISIS 420915	TCTTGGTTACATGAAATCCC	20-mer	2′MOE gapmer PS backbone	7,183.24	transthyretin amyloidosis
4	ISIS 681257	TGCTCCGTTGGTGCTTGTTC	20-mer	2′MOE gapmer PS/PO mixed- backbone conjugated to 5′trishexylamino-C_6_-GalNAc_3_	8,636.47	coronary artery disease

GalNAc_3_, triantennary N-acetyl galactosamine; 2′MOE, methoxyethyl; PO, phosphodiester; PS, phosphorothioate.

## Results

### CYP1A2, CYP2B6, CYP2C8, CYP2C9, CYP2C19, CYP2D6, CYP2E1, and CYP3A4 Enzyme Inhibition

The incubation conditions were optimized with acceptable dosing concentrations (usually at Michaelis constant [K_m_]) and respective signal levels for detection. Incubation times of 45 or 90 min were selected across isoforms using known probe substrates, and the same time interval was applied to both the positive control and antisense drug test articles. Due to the slow metabolism process of ASOs, no major differences were observed between 45 and 90 min based on pilot experiments. Two studies were done at different incubation times and results were reported. For a single ASO (ISIS 396443), the study was done at a different date using a different concentration unit, μg/mL, as opposed to μM. Overall, a lower concentration was used because the clinical dose of the drug was lower, 12 mg by intrathecal route, and the expected systemic exposure is much less in comparison to subcutaneous delivery.

**Figure 1:**
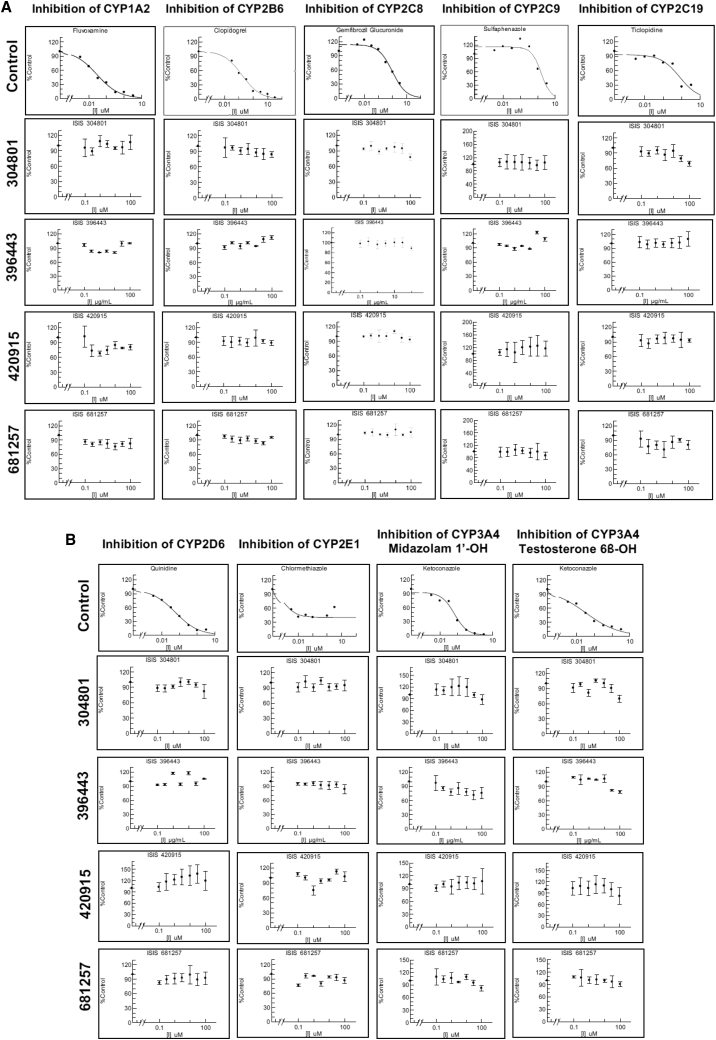
CYP Inhibition Studies (A and B) CYP1A2, CYP2B6, CYP2C8, CYP2C9, and CYP2C19 (A) and CYP2D6, CYP2E1, CYP3A4 (midazolam 1′-hydroxylation), and CYP3A4 (testosterone 6β-hydroxylation) (B) enzyme activity by ISIS 304801, 396443, 420915, 681257, positive control, and vehicle. Data for ASOs represent the mean fold inhibition of triplicate analysis ± corresponding SD. Human hepatocytes were pre-incubated with CYP-inhibitor-positive control or ASO solutions were followed by the addition of CYP isoenzyme-specific substrates for 30 min using a 45-min incubation time.

The half maximal inhibitory concentration (IC_50_) values of three 2′-MOE-modified ASOs, ISIS-304801, ISIS-396443, and ISIS-420915, for CYP1A2, CYP2B6, CYP2C8, CYP2C9, CYP2C19, CYP2D6, CYP2E1, and CYP3A4 were all greater than 100 μM, 100 μg/mL, and 100 μM, respectively. Similar findings were also observed using a GalNAc_3_-conjugated 2′-MOE-ASO, ISIS-681257. The IC_50_ values of the positive controls ranged from 0.00108 to 1.56 μM, as expected for these CYP enzymes. The IC_50_ data are presented in Table 2; data for the inhibition of CYP enzyme activities by the positive controls are presented in the top row of Figures 1A and 1B, respectively. After the 1-hr incubation, enzymatic activity was well maintained at approximately >70%–130% of the vehicle control for all enzymes studied (Figures 1A and 1B). There was no evidence of a concentration-dependent decrease in CYP1A2, CYP2B6, CYP2C8, CYP2C9, CYP2C19, CYP2D6, CYP2E1, or CYP3A4 enzyme activity after incubating each probe substrate with the respective ASOs. In contrast, the positive controls for each isoenzyme showed potent concentration-dependent inhibition of CYP enzyme activity.

**Table 2:** CYP Inhibition IC_50_ Values from Human Hepatocyte Incubations

Isoenzyme	ASO (μM or μg/mL)	Positive Control	Positive Control (μM)	Incubation Time (min)
CYP1A2	>100	fluvoxamine	0.0287	45
CYP2B6	>100	clopidogrel	0.163	45
CYP2C8	>100	gemfibrozil glucuronide	0.960	90
CYP2C9	>100	sulfaphenazole	1.56	45
CYP2C19	>100	ticlopidine	0.767	90
CYP2D6	>100	quinidine	0.0569	45
CYP2E1	>100	chlormethiazole	0.00108	90
CYP3A4 (midazolam)	>100	ketoconazole	0.0670	45
CYP3A4 (testosterone)	>100	ketoconazole	0.0324	45

Note: ASO concentrations for ISIS 304801, 420915, and 681257 refer to μM, whereas μg/mL is used for ISIS 396443. IC_50_, half maximal inhibitory concentration.

### CYP1A2, CYP2B6, and CYP3A4 Enzyme and mRNA Induction

The enzymatic activity of CYP1A2 was determined by comparing the peak area ratios of acetaminophen to the peak area ratios of internal standard (tolbutamide). The mean data from the CYP1A2 enzyme activity experiment demonstrated that three 2′-MOE-ASOs, ISIS-304801, ISIS-396443, and ISIS-420915, at concentrations of up to 100 μM induced between 0.723-fold and 1.44-fold CYP1A2 enzyme activity. Similar results were observed with ISIS-681257 (a GalNAc_3_-conjugated 2′-MOE-ASO), with a 1.40-fold induction of CYP1A2 enzyme activity, whereas omeprazole at 50 μM induced a 50.6-fold of CYP1A2 enzyme. Mean CYP1A2 mRNA data showed a 0.625-fold to 2.01-fold induction for unconjugated 2′-MOE-ASOs, a 1.71-fold induction for the GalNAc_3_-conjugated ASO, and a 45.7-fold induction for omeprazole.

The enzymatic activity of CYP2B6 was determined by comparing the peak area ratios of hydroxyl-bupropion to the peak area ratios of tolbutamide. The mean data from the CYP2B6 enzyme activity experiment demonstrated that unconjugated 2′-MOE-ASOs at up to 100 μM induced between 0.634-fold and 1.84-fold of CYP2B6 enzyme activity. Similarly, the GalNAc_3_-conjugated ASO induced 1.20-fold of CYP2B6 enzyme activity, whereas phenobarbital induced 5.03-fold of CYP2B6 enzyme activity. Mean CY2B6 mRNA data showed fold inductions ranging from 0.604 to 1.32 for unconjugated 2′-MOE-ASOs. Similarly, a 1.36-fold induction in CYP2B6 mRNA was observed for the GalNAc_3_-conjugated ASO in contrast to a 9.83-fold induction after treatment with phenobarbital.

The enzymatic activity of CYP3A4 was determined by comparing the peak area ratios of hydroxyl-midazolam to the peak area ratios of tolbutamide. The mean data from the CYP3A4 enzyme activity experiment demonstrated that unconjugated 2′-MOE-ASOs at up to 100 μM induced between 0.725-fold and 2.28-fold of CYP3A4 enzyme activity, whereas the GalNAc_3_-conjugated ASO induced 1.67-fold of CYP3A4 enzyme activity. In contrast, rifampicin at 10 μM induced 9.14-fold of CYP3A4 enzyme activity. Mean CYP3A4 mRNA data showed a fold induction ranging from 1.01 to 1.35 for unconjugated 2′-MOE-ASOs, whereas the GalNAc_3_-conjugated ASO induced 0.213-fold of CYP3A4 mRNA, in contrast to a 4.97-fold induction after treatment with rifampicin. As such, no evidence of a significant increase in CYP1A2, CYP2B6, or CYP3A4 enzyme activity or mRNA was demonstrated after treatment with any of the four ASOs at concentrations of up to 100 μM (Table 3).

**Table 3:** Fold Induction of CYP1A2, CYP2B6, and CYP3A4 Enzyme Activity and mRNA by Antisense Oligonucleotides and Respective Controls

Isoenzyme	Assay	Positive Control	304801	396443	420915	681257
CYP1A2	enzyme activity	50.6 ± 3.78	1.44 ± 0.137	0.751 ± 0.206	0.723 ± 0.142	1.40 ± 0.116
	mRNA	45.7 ± 4.51	2.01 ± 0.111	0.625 ± 0.219	1.88 ± 0.449	1.71 ± 0.0853
CYP2B6	enzyme activity	5.03 ± 1.06	1.84 ± 0.199	0.634 ± 0.0213	1.40 ± 0.314	1.20 ± 0.389
	mRNA	9.83 ± 0.992	1.32 ± 0.106	0.604 ± 0.382	1.05 ± 0.200	1.36 ± 0.347
CYP3A4	enzyme activity	9.14 ± 0.419	2.28 ± 0.200	0.725 ± 0.0465	1.33 ± 0.164	1.67 ± 0.310
	mRNA	4.97 ± 0.445	1.35 ± 0.0382	1.12 ± 0.195	1.01 ± 0.00399	0.213 ± 0.0471

Note: Data represent the mean fold induction of triplicate analysis ± corresponding SD. Respective ASO concentrations were 100 μM, positive controls included 50 μM of omeprazole (CYP1A2), 1,000 μM of phenobarbital (CYP2B6), and 10 μM rifampicin of (CYP3A4). Human hepatocytes were incubated for 72 hr. Vehicle control applied for positive controls was DMSO at 0.5%, whereas for antisense drugs, PBS at 1% was used.

There was a slight reduction in fold induction for 396443 (at 100 μM) relative to its vehicle control (<2-fold) across all isoenzymes. A potential possibility was that there were slight cell damages at a high concentration of 396443, causing a slight loss of mRNA and enzyme activities.

### Hepatocyte Uptake

**Figure 2:**
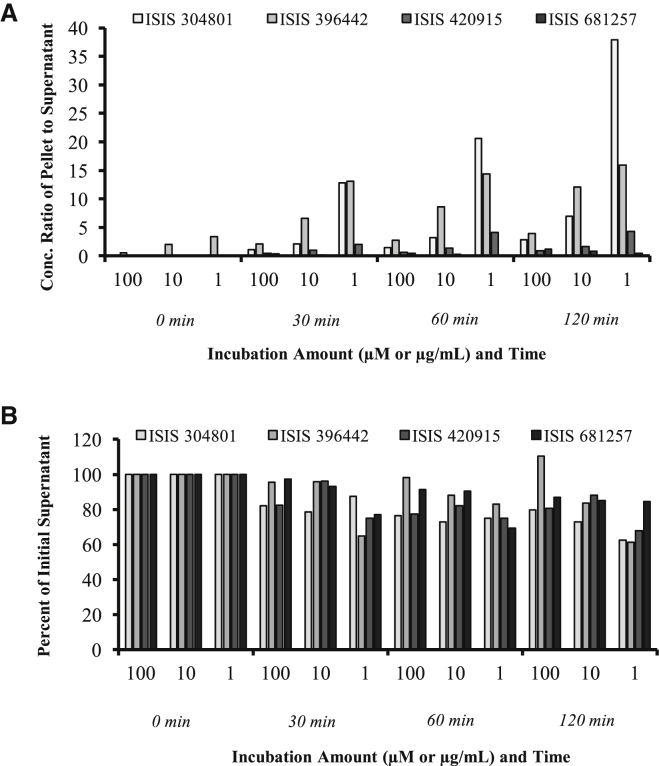
Cell Uptake Summary Uptake of ISIS 304801, ISIS 396443, ISIS 420915, and ISIS 681257 in human hepatocytes. (A and B) Concentration ratio in pellet normalized to supernatant amount at time 0 (A) and percent of supernatant normalized to time 0 (B). Incubation amount refers to μM for ISIS 304801, 420915, and 681257 and μg/mL for ISIS 396443, respectively. Samples, curves, QCs, and internal standard were processed by liquid-liquid extraction and solid phase extraction prior to analysis by LC-UV/MS. Two calibration curves were used, with the linear range for LC-MS and LC-UV/Vis as 0.064–641.03 μM and 32.05–4,807.69 μM, respectively.

Hepatocyte uptake experiments demonstrated rapid and sufficient accumulation of ASOs within hepatocytes, with average cellular concentrations ranging from 56.3 to 140 μM for ISIS-304801, 26.5 to 50.5 μM for ISIS-396443, and 52.2 to 106 μM for ISIS-420915 after a 30- and 120-min incubation time, respectively. Similar results were obtained with ISIS 681257, the GalNAc_3_-conjugated ASO: 14.0 to 50.6 μM after 30 and 120 min, respectively. Cell uptake of ISIS 304801, ISIS 396443, ISIS 420915, and ISIS 681257 in human hepatocytes was summarized with the concentration ratio of ASO in the pellet normalized to the supernatant amount at time 0 (Figure 2A) and included a plot of the percentage of each supernatant normalized to time 0 (Figure 2B). A dose-dependent uptake within the hepatocyte pellet was observed, with substantially greater uptake proportions compared to supernatant present at the lower doses. Concentrations in the hepatocyte increased over the 120-min incubation interval.

Of note, the cellular concentrations of ISIS 681257 at a test concentration of 1.00 μM was below the level of quantitation at time points 0–60 min, but reached a concentration of 0.626 μM after 120 min of incubation. Average concentrations of ASOs in cell supernatants following incubation with 100 μM of each ASO ranged from 38.2 to 50.0 μM for ISIS 304801, 12.1 to 14.0 μM for ISIS 396443, and 85.7 to 111 μM for ISIS 420915 after a 0- to 120-min incubation time. Similar results were obtained with ISIS 681257, the GalNAc_3_-conjugated ASO: 37.5 to 43.1 μM after 0 to 120 min. Concentrations of ASOs in cell supernatants were dose proportional between 1 and 100 μM. The cellular uptake experiment in this study demonstrated that under the CYP inhibition assay conditions, the cellular concentrations of each of the ASOs tested were proportional to the concentrations tested. The highest concentration associated with hepatocytes in this study was approximately 5- to 10-fold higher than our estimates of clinically relevant accumulations in the liver.

### Substrate Uptake Studies of Major SLC and ABC Transporters

**Figure 3:**
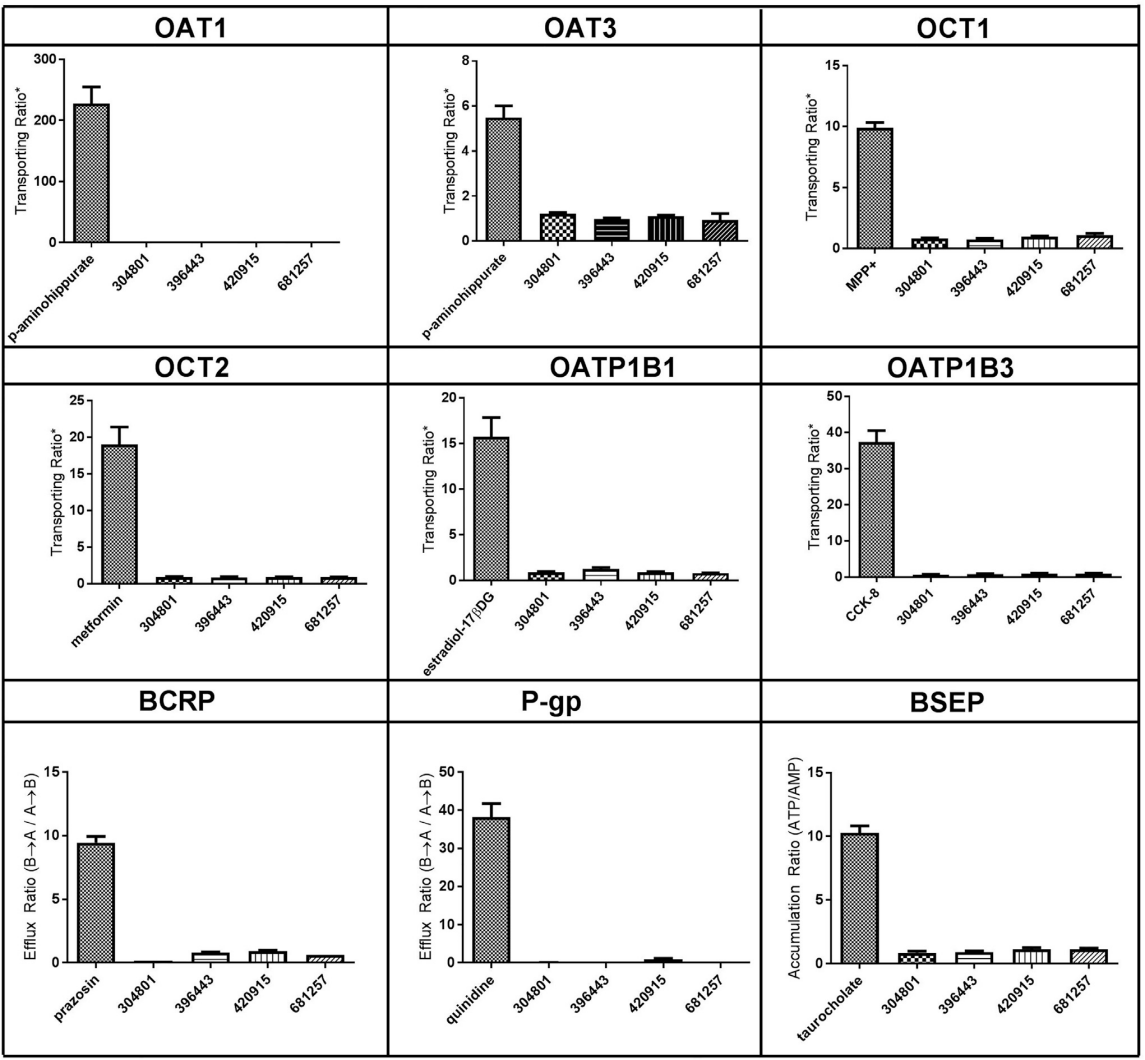
In Vitro Uptake Mediated by SLC and ABC Transporters For all transporters except BSEP, the cell line was MDCK. BSEP was studied in membrane vesicles prepared from Sf9 cells. Transporting ratio is the signal to noise for SLC transporters, as defined by the ratio of substrate transported in transporter-expressed cells/non-expressed cells. The concentration of each ASO, including ISIS 304801, ISIS 396443, ISIS 420915, and ISIS 681257 was studied at 10 μM. Positive control substrates for each transporter included 2 μM p-aminohippurate (OAT1), 10 μM p-aminohippurate (OAT3), 2 μM MPP^+^ (OCT1), 10 μM metformin (OCT2), 2 μM estradiol-17-β-D-glucuronide (OATP1B1), and 2 μM CCK-8 (OATP1B3). Efflux ratio is the P_app_ in the B to A direction divided by the P_app_ in the A to B direction. Accumulation ratio is the signal to noise for the BSEP transporter, as defined by the vesicular accumulation (ATP)/vesicular accumulation (AMP). The concentration of each ASO, including ISIS 304801, ISIS 396443, ISIS 420915, and ISIS 681257, was studied at 10 μM. Positive control substrates for each transporter included 2 μM prazosin (BCRP), 100 nM quinidine (P-gp), and 1 μM taurocholate (BSEP). Data represent the mean and SD of triplicate samples.

The positive controls for all transport assays satisfied respective control criteria. At a concentration of 10 μM unconjugated 2′-MOE-ASO (ISIS 304801, 396443, and 420915) or GalNAc_3_-conjugated ASO (ISIS 681257), the OAT1-, OAT3-, OCT1-, OCT2-, OATP1B1-, OATP1B3-, and BSEP-mediated uptake of each ASO did not show 2-fold greater uptake than that in the corresponding controls (Figure 3), indicating that none of these ASOs (both unconjugated and GalNAc_3_ conjugated) tested at 10 μM were substrates for any of the transporters tested. At a concentration of 10 μM ISIS 304801, 396443, 420915, or 681257, the efflux ratios were not above 2.0 for either BCRP or P-gp efflux transporters (Figure 3), indicating that these 4 ASOs were not substrates of either BCRP or P-gp. The mass balance of ASOs in the BCRP and P-gp assays included ISIS 304801, ranging from 94.3% to 138%, ISIS 396443 (81.3%–99.5%), and ISIS 420915 (102%–129%), and were similar with ISIS 681257 (78.0%–91.0%), indicating good recovery of all ASOs.

### Inhibition Studies of Major SLC and ABC Transporters

**Figure 4:**
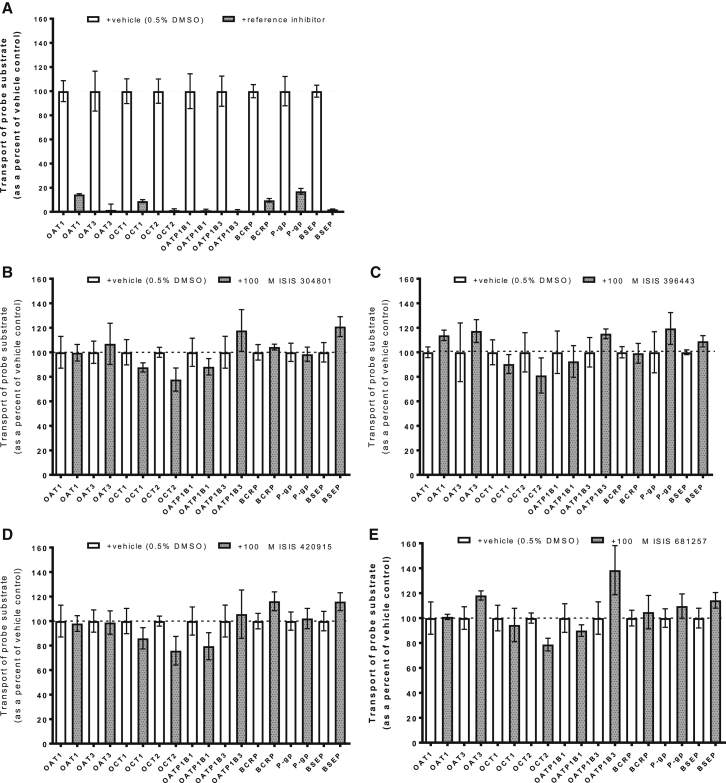
Inhibition of SLC and ABC Transporter-Mediated Probe Substrate Transport (A–E) Inhibition of positive controls (A), ISIS 304801 (B), ISIS 396443 (C), ISIS 420915 (D), and ISIS 681257 (E). For all transporters except BSEP, the cell line was MDCK. BSEP was studied in membrane vesicles prepared from Sf9 cells. The concentration of reference inhibitor probenecid was 100 μM (OAT1) (OAT3), quinidine was 1,000 μM (OCT1) (OCT2), rifampicin was 100 μM (OATP1B1) (OATP1B3) or 300 μM (BSEP), Ko143 was 1 μM (BCRP), and elacridar was 3 μM (P-gp). The concentration of each ASO was studied at 100 μM. Probe substrates for each transporter included 2 μM p-aminohippurate (OAT1), 10 μM p-aminohippurate (OAT3), 2 μM MPP^+^ (OCT1), 10 μM metformin (OCT2), 2 μM estradiol-17-β-D-glucuronide (OATP1B1), 2 μM CCK-8 (OATP1B3), 2 μM prazosin (BCRP), 100 nM quinidine (P-gp), and 1 μM taurocholate (BSEP). Data represent the mean and SD of triplicate samples. Dashed line = 100%. A highly statistically significant inhibition was obtained for all positive controls (p < 0.001).

The positive controls for all inhibition assays satisfied respective control criteria. IC_50_ values were determined for each reference inhibitor, and reference inhibitors were used at concentrations of ≥10X the IC_50_ value, which corresponded to ≥85% inhibition. The inhibitory effects of ISIS 304801, 396443, 420915, and 681257 on transport of substrate by OAT1, OAT3, OCT1, OCT2, OATP1B1, OATP1B3, BCRP, P-gp, and BSEP was investigated. The transport of substrate in the presence of the vehicle control (water) was compared to the uptake in the presence of test ASO or a known reference inhibitor. As a positive control reference inhibitor, probenecid (100 μM) was shown to inhibit up to 85.7 ± 0.843% and 98.3 ± 6.07% OAT1- and OAT3-mediated transport of p-aminohippurate, respectively (Figure 4A). Similarly, quinidine (1,000 μM) inhibited 91.2 ± 1.57% of OCT1-mediated transport of MPP^+^ and 98.5 ± 1.34% of OCT2-mediated transport of metformin. Rifampicin (100 μM) inhibited 98.8 ± 1.09%, 99.0 ± 0.955%, and 98.0 ± 0.382% of OATP1B1-, OATP1B3-, and BSEP-mediated transport of estradiol-17β-D-glucuronide, CCK-8, and taurocholate, respectively. Finally, Ko143 inhibited 90.4 ± 1.89% of BCRP-mediated transport of prazosin, and elacridar inhibited 90.2 ± 1.42% of P-gp-mediated transport of quinidine (Figure 4A).

Using unconjugated 2′-MOE-ASOs at a concentration of 100 μM (ISIS 304801, 396443, and 420915), the mean % inhibition ranged from −21.0% to 22.3%, −19.4% to 19.0%, and −16.2% to 24.2% for all transporters evaluated, respectively. Similar results were obtained for the GalNAc_3_-conjugated ASO, ISIS 681257, whereby the mean % inhibition ranged from −38.3% to 21.3% for all transporters, respectively (Figures 4B–4E). Very slight enhancement of probe substrate uptake in the presence of 100 μM ISIS 304801 or 396443 was observed for BSEP: 21.0% (p = 0.0329) and 8.99% (p = 0.0362), respectively. Minimal enhancement of probe substrate uptake in the presence of 100 μM ISIS 396443 was observed for OAT1 (13.8%, p = 0.0167). Lastly, minimal enhancement of probe substrate uptake in the presence of 100 μM ISIS 681257 was observed for OAT3 (18.2%, p = 0.0326) and OATP1B3 (38.3%, p = 0.0476). The absolute magnitude of these apparent enhancements are smaller than the magnitude of enhancement or inhibition for several of the other transporters tested and is most likely due to random chance. Because the concentration of these ASOs (100 μM) significantly exceed peak plasma concentrations (C_max_) of relevant clinical doses, it is highly unlikely that this marginal increase in transport is clinically relevant. ISIS 304801, 396443, 420915, and 681257 are not considered inhibitors of either BCRP, P-gp, OAT1, OAT3, OCT1, OCT2, OATP1B1, OATP1B3, or BSEP.

## Discussion

It is well established that the major CYP enzymes and drug transporters make a significant contribution to the PK and pharmacodynamic (PD) properties of small molecules, yet the interaction and relation of unconjugated ASOs toward these enzymes and drug transporters are not as well characterized, with limited DDI data and no existing investigations of GalNAc_3_-ASO conjugates. Antisense drugs may be used within very diverse treatment areas, and, as such, an ASO therapy requiring the combination of several concomitant medications as a standard of care may be encountered frequently. The modulation of CYP enzymes and transporters by DDIs has been shown to define systemic and tissue concentrations of small molecules, leading to inter-individual treatment variability and even adverse accounts of increased toxicity and mortality for several molecules.^26^ Therefore, a complete knowledge of the enzymatic pathway of ASOs and the potential interactions with CYP enzymes and drug transporters are of major importance to ensure treatment safety and efficacy.

Because ASOs are drastically different from small molecules in their physico-chemical properties, such as molecular weight, number of hydrogen bonds, and disposition, the ability to interact with CYP enzymes and drug transporters is significantly limited. Similarly, sequence and secondary structure of ASOs are unlikely making a difference for ASO-CYPs or ASO-transporter interactions at the protein level (as a substrate or inhibitor) because three-dimensional structures of ASOs are so similar among different sequences and modifications. At the RNA level, sequence or secondary structure could make a difference theoretically if the sequence of an ASO happens to hybridize with the RNA of a CYP or transporter in the nucleus. However, the probability of a perfect match and hybridization with the RNAs of CYPs or transporters on top of a perfect match with the target RNA of the disease is unlikely or remote for 20-mer ASOs with careful designs.

ASOs are readily taken up into numerous types of liver cells, including parenchymal, non-parenchymal, and sinusoidal endothelial cells, and have long been known to be metabolized into shortmers by endonucleases and exonucleases without being subjected to metabolism by CYP enzymes. More recently, GalNAc_3_-ASO-conjugation strategies have offered hepatic-specific internalization of ASOs in a target-mediated disposition process. The major pathways for cellular uptake of antisense oligonucleotides are much different from small molecules and are presumed to be by endocytosis and involve the interaction with proteins on the cell surface. ASOs modified with phosphorothioate linkages stick to cell surface proteins and internalize into cells at the cell surface; the protein is internalized by endocytosis or membrane turnover. Recently published data have shown that the asialoglycoprotein receptor (ASGR), along with other cell surface proteins, are involved in the uptake of GalNAc conjugated and unconjugated ASOs. These receptors have been shown to be expressed in human hepatocytes in vitro.^27^ Because CYP enzymes are membrane-bound and located in the endoplasmic reticulum, the CYP inhibition potentials may be impacted by subcellular concentrations during the probe substrate incubation and by compartmentalization after the drug enters the cells.

The intention of the in vitro CYP- and/or transporter-mediated DDI studies was to evaluate the potential of DDI in vivo at exceedingly high exposure scenarios. Incubation concentrations for three compounds included a high concentration at 100 μM, which is several fold higher than the projected liver exposures in humans or monkeys at clinically relevant doses (because the monkey is a good PK model for man based on mg/kg dose).^14^, ^28^, ^29^ ASOs accumulate extensively in tissues (especially in the liver and kidney), where DDIs may take place, if any, at these high concentrations. Similarly, for transporter substrate evaluation, a 10-μM concentration was selected to cover the maximum observed plasma exposures (C_max_) at clinically relevant doses to ensure the maximum uptake or transport of the drug can be seen in different transporter studies because the overall uptake or transport of ASO is a slow process in all cell lines/systems. The distribution of ASOs is a rapid process, and C_max_ represents the concentration of the drug that could potentially interface with the transporter proteins. The C_max_ concentrations are normally ∼1 μM following subcutaneous (s.c.) dosing and could be several fold higher after intravenous (i.v.) bolus injection.^18^ Under these extreme experimental conditions, results in vitro can be extrapolated to in vivo with confidence; however, further in vivo studies may still be necessary.

The hepatocyte uptake experiment was performed to ensure appropriate uptake during the treatment time (as optimized by controls). The maximum range of uptake into hepatocytes is several fold greater than projected liver exposure. The transporter uptake and inhibition and CYP inhibition and uptake assays were carefully optimized before screening each antisense drug. Measured cellular concentrations of ASOs in the hepatocyte uptake arm of the experiment demonstrated that sufficient concentrations of ASOs accumulated in the hepatocytes during the 120-min incubation. Concentrations in the supernatant were comparable across all compounds and showed a decreasing trend with time, as shown in Figure 2, suggesting that there was a fraction of drug distributed into the pellet. However, in the pellet, there appeared to be differences in concentrations (as shown as pellet/supernatant ratios), even among the three unconjugated ASOs. ISIS 304801 and ISIS 396442 showed similar patterns, whereas ISIS 420915 was lower. The exact mechanistic reason for these differences is unknown. For the conjugated ASO (ISIS 681257), a higher distribution into tissue (compared to unconjugated) was expected based on the mechanism of tissue uptake and data reported previously.^13^, ^30^, ^31^ To the opposite, the pellet exposure was the lowest for ISIS 681257, which was likely due to liquid chromatography-mass spectrometry (LC/MS) analytical issues, in which only the parent drug molecular weight (MW) for the ASO was monitored. There is a likelihood of metabolism, including sugar deletion that had occurred after the drug was taken up in cells. ISIS 681257 metabolites, including ISIS 681257-1GalNAc, ISIS 681257-2GalNAc, and ISIS 681257-3GalNAc sugars, were not quantitated at the time of analysis and could have resulted in an underestimated exposure. Further studies are warranted to confirm these findings. For the three unconjugated ASOs, differences in uptake, as measured in the pellet, may translate into different outcomes in vivo, including tissue concentration and PK/PD profiles. However, in vivo liver exposure, as observed in monkey toxicokinetic studies, showed that all four ASOs, including the GalNAc-conjugated ASO, had comparable dose-normalized liver concentrations (within ∼2-fold of each other based on internal unpublished data), indicating that the in vitro uptake results may not translate appropriately to in vivo.

Results of the CYP inhibition study demonstrated that none of the ASOs tested (either unconjugated or GalNAc_3_ conjugated) are an inhibitor of CYP1A2, CYP2B6, CYP2C8, CYP2C9, CYP2C19, CYP2D6, CYP2E1, or CYP3A4 enzyme activity when incubated at concentrations of up to 100 μM (ISIS 304801, 420915, and 681257) or up to 100 μg/mL for ISIS 396442, with prototypical CYP substrates in suspensions of cryopreserved human primary hepatocytes. The concentrations tested were several fold higher than those expected in clinical studies. Therefore, DDI of the 2′-MOE-modified ASOs with co-administered small molecule drugs on the CYP P450 level, either as inducers or inhibitors, is unlikely. These findings are consistent with previous in vitro CYP inhibition studies using Mipomersin (a commercialized ASO) and were also confirmed in vivo in dedicated clinical DDI studies.^20^

Results of the CYP induction study demonstrated that ISIS 304801, 420915, and 681257 at concentrations of up to 100 μM and ISIS 396442 at a concentration of up to 100 μg/mL did not cause elevation of the enzymes CYP1A2, CYP2B6, or CYP3A4 at either the enzyme activity level or mRNA level in cryopreserved primary human hepatocytes. Under the same experimental conditions, the prototypical inducers of CYP1A2, CYP2B6, and CYP3A4 showed significant increases at both the enzyme activity level and mRNA level, demonstrating that the experiments were performed appropriately. As well documented in the literature, the in vitro characterization of the main enzyme(s) involved in the metabolism of antisense oligonucleotides reveal that nucleases are responsible for ASO metabolism. Following distribution into the liver, ASOs are slowly metabolized by exonucleases and endonucleases, forming chain-shortened metabolites, which are less plasma protein bound and readily excreted in urine.^32^ Hence, the metabolic pathway and fate of ASOs have been established as CYP-independent.

For the major drug transporters, we found that four different antisense oligonucleotides were neither substrates nor inhibitors across a range of major drug transporters in vitro. Collectively, several ASOs, including a GalNAc_3_-conjugated ASO, have now been extensively screened in the regulatory recommended drug-transporter panel and made available as a reference. These studies serve as an extensive expansion and confirmation of our understanding of the role and lack of interactions with the major drug transporters in ASO therapy and as a complement to data on the lack of transporter interactions with ISIS 141923.^33^

In conclusion, this research is important in summarizing up to date knowledge of the lack of ASO transporter and ASO-CYP interplay using a marketed compound as well as several development compounds and allows a better understanding of the lack of ASO-transporter interactions and lack of CYP metabolism in ASO disposition. No DDI on the major CYP and transporter level was observed across any of the four antisense drugs evaluated in the present study; however, in vitro drug interaction studies may still be recommended for ASOs coming into development to ensure safety concerns. Our investigations suggest a low risk for DDIs with 2′MOE-ASOs and confer flexibility in the therapeutic use of antisense medicines with concomitant drugs without the need for dose adjustment. Our observations are in line with several previous preclinical and clinical investigations for ASO therapeutics and provide additional insights for 2′-MOE-ASO-GalNAc_3_ conjugates.

## Materials and Methods

### Materials

Hank’s balanced salt solution (HBSS), pH 7.4, without phenol red, PBS, DMEM, and 10% fetal bovine serum (FBS) were purchased from Corning (Corning, NY). Millicell 96-well insert plate with permeable membrane (PCF –0.4 μm), Millicell 96-well receiver tray, and MultiScreen HTS FB Filter Plate (1.0/0.65 μM) were purchased from Millipore. PerkinElmer 96-well Flex plates, Ultima Gold XR Scintillation Fluid, [^3^H]-MPP^+^ (N-methyl-4-phenylpyridinium), [^3^H]-p-aminohippurate, [^3^H]-estradiol-17b-D-glucuronide, [^3^H]-CCK8, [^3^H]-prazosin, and [^3^H]-taurocholate were purchased from PerkinElmer (Waltham, MA). MDCK-II cells used were a purified subclone of MDCK-II cells that were obtained from the University of California, San Francisco. MDCK-MDR1 cells were obtained as a stably transfected clone at Optivia Biotechnology (Menlo Park, CA). BSEP assay uptake buffer (10 mM 4-(2-hydroxyethyl)-1-piperazineethanesulfonic acid [HEPES]-Tris, pH 7.4, 0.1 M KNO_3_, 12.5 mM Mg(NO_3_)_2_, and 50 mM sucrose), wash buffer (10 mM Tris-HCl, pH 7.4, 0.1 M KNO_3_, and 50 mM sucrose), blocking buffer (BSEP Wash Buffer + 0.5 mg/mL BSA), HEPES, Tris, KNO_3_, MgNO_3_, sucrose, BSA, Williams’ Medium E, acetonitrile, CYP enzymes (1A2, 2B6, 2C8, 2C9, 2C19, 2D6, 2E1, and 3A4), probe substrates (phenacetin, bupropion, paclitaxel, diclofenac, (S)-mephenytoin, dextromethorphan, chlorzoxazone, midazolam, and testosterone), metabolites (acetaminophen, 4-hydroxybupropion, 6α-hydroxypaclitaxel, 4’-hydroxydiclofenac, 4-hydroxymephenytoin, dextrorphan, 6-hydroxychlorzoxazone, 1’-hydroxymidazolam, and 6β-hydroxytestosterone), and inhibitors (fluvoxamine, clopidogrel, gemfibrozil glucuronide, sulfaphenazole, ticlopidine, quinidine, chlormethiazole, and ketaconazole) were purchased from Sigma-Aldrich (St. Louis, Missouri). BSEP Sf9 vesicles and Opti-MEM were purchased from Life Technologies (Carlsbad, CA). 25 mM ATP, 25 mM AMP, MPP^+^ (N-methyl-4-phenylpyridinium), metformin, p-aminohippurate, estradiol-17b-D-glucuronide, prazosin, quinidine, taurocholate, and DMSO were purchased from Sigma. [^14^C]-metformin was purchased from Moravek Biochemicals (Brea, CA). [^3^H]-quinidine was purchased from American Radiolabeled Chemicals (St. Louis, MO). CCK8 was purchased from American Peptide Company (Vista, CA). Cell extraction solution for test article: RTL buffer and QIAGEN RNeasy Mini Kit were purchased from QIAGEN (Valencia, CA). Plated cryopreserved human hepatocytes in a 24-well format, and OptiCulture Hepatocyte Media were purchased from XenoTech, LLC (Lenexa, KS). High-pressure liquid chromatography (HPLC) grade water was obtained in house with a Direct Q-3 water purification system. High-Capacity cDNA Reverse Transcription Kit, MicroAmp EnduraPlate 96- and 384-well reaction plates, primers for human CYP1A2, CYP2B6, CYP3A4, glceraldehyde-3-phosphate dehydrogenase (GAPDH), and TaqMan universal PCR master mix was purchased from Thermo Fisher Scientific (Waltham, MA). Cryopreserved human hepatocytes in suspension were purchased from BioreclamationIVT (Baltimore, MD).

### Test Articles

ISIS 304801 is a 20-mer (5-10-5) 2′-O-methoxyethyl-modified phosphorothioate antisense inhibitor of apolipoprotein C-III (apoC-III) with a sequence of 5′AGC^M^TTC^M^TTGTC^M^C^M^AGC^M^TTTAT-3′. Underlined bases indicate 2′-O-methoxyethyl modifications, and all cytosines are composed of 5-methyl cytosines (C^M^). ISIS 396443 is an 18-mer uniform modified 2′-MOE-phosphorothioate antisense oligonucleotide, which promotes inclusion of exon-7 in splicing of survival of motor neuron 2 (SMN 2) pre-messenger RNA for production of full-length SMN protein. ISIS 420915 is a 20-mer 2′-O-methoxyethyl-modified phosphorothioate antisense oligonucleotide inhibitor of transthyretin (TTR), with a sequence of 5′TC^M^TTGGTTAC^M^ATGAAATC^M^C^M^C^M^-3′. Lastly, ISIS 681257 is a 20-mer (5-10-5) 2′-O-methoxyethyl-modified mixed phosphorothioate/phosphodiester (PS/PO) backbone antisense oligonucleotide with a 5′-trishexylamino-(THA)-C3GalNAc_3_ endcap designed to target lipoprotein(a) (Lp(a)). All ASOs were synthesized at Ionis Pharmaceuticals (Carlsbad, CA). ASO solution standards corresponding to concentrations of up to 100 mg/mL were stored in a refrigerator set to 2°C–8°C prior to use. Table 1 contains a list of the ASOs used.

### Hepatocyte Inhibition

Preincubations were performed in 50 μL of Williams’ Medium E with 2 mM salicylamide. The salicylamide was included to reduce possible phase II conjugation to the precursor phase I metabolites. Serially diluted CYP-inhibitor-positive control solutions were prepared at final concentrations of 0, 0.00330, 0.0100, 0.0330, 0.100, 0.330, 1.00, and 3.30 μM for CYP1A2, CYP2C8, CYP2C9, CYP2C19, CYP2D6, CYP2E1, and CYP3A4; and 0, 0.0330, 0.100, 0.330, 1.00, 3.30, 10.0, and 33.0 μM for CYP2B6. ISIS 304801, 420915, and 681257 were serially diluted to final concentrations of 0, 0.100, 0.300, 1.00, 3.00, 10.0, 30.0, and 100 μM. ISIS 396443 was serially diluted to final concentrations of 0, 0.100, 0.300, 1.00, 3.00, 10.0, 30.0, and 100 μg/mL. Human hepatocytes (pooled from 5 donors) were thawed and prepared according to the vendor’s protocol. On a 96-well plate, the hepatocytes (25 μL per well, final concentration of 1 million hepatocytes per mL, viability >75%) were mixed with 25 μL of CYP-inhibitor-positive control or with each respective ASO solution at various concentrations and then preincubated in triplicate at 37°C with 5% CO_2_ for 30 min. Following preincubation, 50 μL of the CYP isoenzyme-specific substrates in Williams’ Medium E (final concentrations: 35 μM phenacetin [CYP1A2], 100 μM bupropion [CYP2B6], 10 μM paclitaxel [CYP2C8], 8 μM diclofenac [CYP2C9], 50 μM (*S*)-mephenytoin [CYP2C19], 10 μM dextromethorphan [CYP2D6], 60 μM chlorzoxazone [CYP2E1], 2.5 μM midazolam [CYP3A4], and 50 μM testosterone [CYP3A4]) were then added to start the CYP activity assays. Incubations were carried out at 37°C with 5% CO_2_. The incubation time was 45 min for phenacetin, diclofenac, dextromethorphan, midazolam, bupropion, chlorzoxazone, and testosterone and 90 min for paclitaxel and (*S*)-mephenytoin. The reactions were stopped by adding 200 μL of ice-cold acetonitrile (can) with tolbutamide (100 ng/mL) as the internal standard to 100 μL of hepatocyte suspension (1:2, v/v). The 96-well plate was then vortexed at 1,700 rpm for 3 min and centrifuged at 3,500 rpm for 10 min at 20°C. The supernatants were analyzed using liquid chromatography-tandem MS (LC-MS/MS).

### Hepatocyte Induction

Upon receipt, sandwich-cultured cryopreserved human hepatocytes (with Matrigel) plated on collagen-coated 24-well plates were inspected under a microscope for general morphology and viability. Fresh culture medium (OptiCulture Hepatocyte Media) was added to each plate that passed the visual inspection. The plates were then placed in an incubator set to 37°C with 5% carbon dioxide (CO_2_) for overnight acclimation. The next morning, and each day thereafter for 3 additional days, the cells were visually inspected, and the culture medium in each of the wells was replaced with fresh culture medium containing 0, 1.00, 10.0, 30.0, or 100 μM of ISIS 304801, 420915, and 681257, and 0, 1.00, 10.0, 30.0, or 100 μg/mL of ISIS 396443 in triplicate, as appropriate. During this time, cells in parallel wells were also treated with 50.0 μM omeprazole (inducer of CYP1A2), 1,000 μM phenobarbital (inducer of CYP2B6), or 10.0 μM rifampicin (inducer of CYP3A4) in triplicate, respectively.

### Enzyme Activity and mRNA Determinations

Following the induction treatment, the cells were washed to remove the dosing solutions and CYP-selective substrates were added to the wells. Prototypical enzyme substrate solutions of 100 μM phenacetin (CYP1A2), 250 μM bupropion (CYP2B6), or 10.0 μM midazolam (CYP3A4) were added in triplicate to different wells of the plates, as appropriate. The plates were then incubated at 37°C with 5% CO_2_ for 60 min. Following incubation, aliquots of the supernatants were transferred from the culture plate to a 96-well collection plate, and an equal volume of tolbutamide in acetonitrile (100 ng/mL) was added to each well as the internal standard. The collection plate was centrifuged at 3,500 rpm for 10 min at 20°C. Aliquots of the supernatants were analyzed by LC-MS/MS for the formation of acetaminophen, hydroxyl-bupropion, or hydroxyl-midazolam as markers for enzyme activity of CYP1A2, CYP2B6, and CYP3A4, respectively.

After collection of the supernatant for catalytic analysis, the remaining cells were washed and lysed with QIAGEN Buffer RLT from the QIAGEN RNeasy Kit. Total RNA was isolated using the QIAGEN RNeasy Kit, and cDNA was synthesized using the Applied Biosystems High-Capacity cDNA Reverse Transcription Kit. PCRs were performed on the Applied Biosystems QuantStudio 7 Flex Real-Time PCR System (Thermo Fisher Scientific, Waltham, MA) using TaqMan assay-based detection. Measurements of gene expression for CYP1A2, CYP2B6, and CYP3A4 were performed using the ΔΔC_Ƭ_ relative gene expression analysis method.

### Hepatocyte Uptake

Hepatocyte suspensions were incubated with ISIS 304801, ISIS 420915, and ISIS 681257 at 1.00, 10.0, and 100 μM, and ISIS 396443 at 1.0, 10.0, and 100 μg/mL under the same conditions (i.e., cell density, matrix, and Williams’ Medium E containing 2 mM salicylamide) present in the hepatocyte inhibition experiment. A time-course experiment was conducted in triplicate for each concentration of test compound for 0, 30, 60, and 120 min incubations at 37°C with 5% CO_2_. At the end of each respective time point, the cell suspensions were centrifuged for 8 min at a speed of 50 × *g*, and the supernatant from each sample was collected. The cell pellets were washed by resuspension in Williams’ Medium E, followed by centrifugation at 50 × *g* for 8 min. The supernatant from the wash step was discarded and the remaining cell pellets were kept for analysis. Cell pellets and respective supernatants were measured for the presence of ASO by LC-MS.

### LC-MS Quantitation of ASOs

Samples were analyzed using a tandem LC-UV/MS method. A total of 72 samples per ASO, corresponding to human hepatocyte cells and supernatant fractions, was assayed. Samples, curves, and controls (QCs) were aliquoted into 96-well plates and internal standard was added. Aliquots in 96-well plates were extracted via a liquid-liquid extraction using ammonium hydroxide and phenol:chloroform:isoamyl alcohol (25:24:1). The aqueous layer was then further processed via solid phase extraction (Phenomenex, Strata X SPE), and then dried down under nitrogen and reconstituted in 140 μL of water containing 100 μM EDTA. Samples were then injected onto an Agilent 6130 LC-MS system for analysis. Two curves were created for quantitation, one utilizing UV absorbance at 260 nm and an additional curve using single ion monitoring data from the mass spectrometer. The calibration range using the MS detector was 0.0640–641 μM with liver homogenate, as used to quantitate the concentration in hepatocyte pellet samples. A minimum signal-to-noise ratio of 5:1 was used to distinguish ASO peaks from background noise. The six-point calibration curve using UV absorbance had a range of 32.1–4,808 μM in liver homogenate, with 32.1 μM defining the lower limit of quantitation (LLOQ), as used to quantitate the concentration in the supernatant. Acceptance criteria for calibration and QC standards were set to 85%–115% of nominal values. For the MS, three QC levels were run in triplicate at 1.60 μM, 160 μM, and 481 μM and eight of the nine QCs passed. For the UV, three QC levels were run in triplicate at 160 μM, 481 μM, and 3,205 μM and all QCs passed. All samples were stored at −70°C ± 10°C upon receipt. Hepatocyte cell count conversion to μM liver weight equivalent was calculated by converting the number of hepatocytes per sample to the liver weight equivalent: [(0.1 × 10^6^ cells per sample)/128 × 10^6^ cells/g)] × (1 g/1,000 mg) = 0.78 mg, where 1 g of liver contains 128 × 10^6^ cells/g. Supernatant concentrations are calculated assuming a 100% recovery and that 1 mL William E Medium is equal to 1 g. The calibration range for ASOs in transporter studies was 0.01–10 μM in 100 μL for RLT buffer and 0.005–10 μM in 100 μL for HBSS buffer.

### Data Calculations for Enzyme Activity and mRNA Induction

The activity of each CYP enzyme was measured by comparing the peak area ratio of the corresponding product to the peak area ratio of the internal standard. The fold induction was calculated by dividing the peak area ratio of each individual well from the treatment group by the mean of the peak area ratios obtained from the vehicle control wells. The mRNA level (ΔC_Ƭ_) for each CYP was normalized to the respective mRNA signal of GAPDH from each well, including the vehicle control wells. The ΔC_Ƭ_ was calculated as follows: ΔC_Ƭ_ = C_Ƭ (CYP)_ − C_Ƭ (GAPDH)_. The relative mRNA levels (ΔC_Ƭ_) were further normalized to the values expressed by the vehicle control wells to obtain the comparative mRNA levels (ΔΔC_Ƭ_). The ΔΔC_Ƭ_ was calculated as follows: ΔΔC_Ƭ_ = Δ C_Ƭ (Treatment)_ − Δ C_Ƭ (Vehicle)_. The fold induction of the target CYP was calculated using the following equation:

$$Fold=2^{\text{-}\Delta \Delta \text{CT}}.$$

### Cell Culture

MDCK-II cells were maintained in DMEM with low glucose and 10% FBS. Cell passages up to 40 were seeded at 60,000 ± 10,000 cells/well onto 96-well transwell membrane plates approximately 24 hr before transfection. All transport assays were carried out approximately 48 hr after transfection. MDCK-MDR1 cells were maintained in DMEM with low glucose, G418, and 10% FBS. Cells between passages 5 and 90 were seeded at 60,000 ± 10,000 cells/well onto 96-well, transwell membrane plates. Transport assays were carried out approximately 72 hr after seeding.

### Transport Study for the Solute Carrier Transporters

A 96-well cell culture plate containing a monolayer of MDCK-II cells grown on a permeable support and a corresponding receiver tray was used. The basolateral compartment of the culture plate and wells were washed with warm HBSS three times, with wells of the culture plate serving as the apical compartment. The wash was aspirated by adding 150 μL of 37°C HBSS to both compartments. Plates were incubated at 37°C with orbital shaking at 60 rpm for 15 min for OAT1, OAT3, OCT1, OCT2, OATP1B1, and OATP1B3 (±5 min). The pre-incubation buffer from both compartments was aspirated and 150 μL of the following was added to the basal compartment. For the probe substrate transport assay, HBSS in each well contained one of following probe substrates (positive control for transport), OCT1, 2 μM [^3^H]-MPP^+^; OCT2, 10 μM [^14^C]-metformin; OAT1, 2 μM [^3^H]-p-aminohippurate; OAT3, 10 μM [^3^H]-p-aminohippurate; OATP1B1, 2 μM [^3^H]-estradiol-17 β-D-glucuronide; and OATP1B3, 2 μM [^3^H]-cholecystokinin octapeptide (CCK-8). For the positive control inhibition assay, HBSS contained one of the following reference inhibitors along with the above probe substrates: OCT1, 1,000 μM quinidine; OCT2, 1,000 μM quinidine; OAT1, 100 μM probenecid; OAT3, 100 μM probenecid; OATP1B1, 100 μM rifampicin; and OATP1B3, 100 μM rifampicin. Inhibition studies contained HBSS in each well, the probe substrate, and 100 μM of each test article, whereas substrate studies contained HBSS in each well and 10 μM of each test article. Plates were incubated at 37°C with orbital shaking at 60 rpm for 5 min. Both sides of the permeable support were washed four times with ice-cold PBS and 60 μL of cell extraction solution was added to each well. Plates were agitated for 15 min at 120 rpm, after which a 30-μL sample of the extract was collected. Samples containing probe substrate were added to 200 μL of scintillation fluid and counted on a 1450 Microbeta (PerkinElmer), whereas samples containing the test article were kept frozen. Test article concentrations, including control samples, were evaluated by an LC-MS method.

### Transport Study for BCRP and P-gp

MDCK-MDR1 cells and MDCK-II cells transfected with BCRP were washed with HBSS at 37°C. Wells of the culture plate served as the apical compartment, and 150 μL of HBSS solutions was added to the apical wells. These solutions also contain one of the following: vehicle (for probe substrate controls), 100 μM test article (for test article inhibition experiments), 1 μM Ko143 (for BCRP inhibition), or 3 μM elacridar (for P-gp inhibition). A 96-well receiver tray was prepared with each well containing 300 μL of buffer solution corresponding to the apical wells. Receiver tray wells served as the basal compartment. The plate and receiver tray were assembled and incubated at 37°C for 30 min. The solution was then aspirated from the apical compartment and the 96-well plate was removed from the receiver tray. Donor and receiver wells for inhibition or donor (containing substrate) and receiver (without substrate) wells for substrate studies were then prepared for bi-directional flux studies. For the probe substrate transport assays, HBSS in each donor well contained one of the following probe substrates: BCRP, 2 μM [^3^H]-prazosin; or P-gp, 100 nM [^3^H]-quinidine. For positive control inhibition assays, HBSS contained the reference inhibitor in both donor and receiver wells, with the probe substrate only in donor wells. For the inhibition assays, including test article assays, HBSS contained the test article in both donor and receiver wells and the probe substrate only in donor wells. For the substrate assays, including test article assays, HBSS in each donor well contained 10 μM test article. For basal to apical (B→A) flux measurements, substrate was placed in the basal compartment only, representing the donor well for B→A flux measurements. The corresponding apical well was devoid of substrate, representing the receiver well for B→A flux measurements. For apical to basal (A→B) flux measurements, substrate was placed in the apical compartment only, representing the donor well for A→B flux measurements. The corresponding basal well was devoid of substrate, representing the receiver well for A→B flux measurements. For substrate studies, an aliquot of ∼400 μL of the dosing solution was saved for mass balance. Both the plate and tray were reassembled and incubated at 37°C with orbital shaking at 50–60 rpm for 90 min. For inhibition studies, a 30-μL sample from each receiver well was obtained, whereas for substrate studies, a 30-μL sample was obtained at the end of the incubation from each receiver well as well as each donor well. For inhibition studies, 200 μL of scintillation fluid was added, whereas for substrate studies, 200 μL of scintillation fluid was added to samples containing probe substrate. These samples were counted on a 1450 Microbeta (PerkinElmer). For samples containing test articles, samples were quantitated using LC-MS.

### Transport Study for BSEP

The test system included a 96-well flat bottom plate containing a suspension of vesicles and a 96-well glass fiber filtration plate. Transport experiments were initiated with the addition of Mg-ATP (for ATP-dependent BSEP uptake) or AMP (non-ATP-dependent uptake) to appropriate wells. Preparation of the flat-bottom plates included incubation with blocking buffer for 60 min at 37°C with orbital shaking at 120 rpm. After 60 min, the blocking buffer was completely removed and assay uptake buffer solutions were added. For BSEP inhibition studies, solutions contained 1 μM [^3^H]-taurocholic acid as a substrate and either assay vehicle, test article (at 100 μM), or reference inhibitor (300 μM rifampicin). For BSEP substrate studies, solutions contained either 1 μM [^3^H]-taurocholic acid or 10 μM test article. Vesicles were added to assay plates and incubated at 37°C with orbital shaking at 120 rpm for 15 min. To initiate the assay, either Mg-ATP or AMP was added to test wells at a final concentration of 5 mM. Plates were incubated at 37°C with orbital shaking at 120 rpm for 15 min. A glass fiber filtration plate was incubated with blocking buffer for 2 min. Vacuum was then applied to remove the blocking buffer, and ice-cold wash buffer was added to the filtration plate. Vesicular transport was quenched by adding ice-cold wash buffer. Assay samples were then mixed as diluted in wash buffer and 175 μL was transferred into the 96-well glass fiber filtration plate to separate vesicles. Filter plates were washed with ice-cold wash buffer and dried under a vacuum. For inhibition studies and for substrate studies that contained probe substrate, filters were transferred to scintillation plates, and 700 μL of scintillation fluid was added. These samples were counted using a 1450 Microbeta (Perkin-Elmer). For substrate study samples containing the test article, filters were transferred to vials and 100 μL of RLT buffer was added with incubation at 37°C with orbital shaking at 60 rpm for 1 hr. The vials were centrifuged, and 100 μL of supernatant was saved into a 96-well plate, which was stored at −80°C. The test article was quantitated by LC-MS.

### Transport Data Analysis and Calculations

Net-transporter-mediated uptake of substrate by each SLC transporter, including OAT1, OAT3, OCT1, OCT2, OATP1B1, and OATP1B3, was calculated by subtracting uptake in the control system, which did not express the transporter of interest, from uptake in the test system, which expressed the transporter of interest according to the equation:

$$\mathrm{Net}\;\mathrm{Transporter}\;\mathrm{Mediated}\;\mathrm{Substrate}\;\mathrm{Uptake}\left(\mathrm{pmol}/\min/cm^{2}\right)=\left(\text{Cellular}\;\text{accumulation}\;\text{in}\;\text{the}\;\text{presence}\;\text{of}\;\text{the}\;\text{transporter}\right)-\left(\text{Mean}\;\text{cellular}\;\text{accumulation}\;\text{in}\;\text{the}\;\text{presence}\;\text{of}\;\text{the}\;\text{transporter}\right).$$

Signal to noise for probe substrates was calculated by dividing the uptake of the probe substrate in transporter-expressing cells by the uptake of probe substrate in control cells not expressing the transporter of interest. Percent inhibition was calculated by dividing the net-transporter-mediated substrate uptake in the presence of the test article or the reference inhibitor by the net-transporter-mediated substrate uptake in the absence of the inhibitor according to the equation:

$$\%\;\text{inhibition}=100-\left(100\times {\left(\text{transporter}\text{-}\text{mediated}\;\text{uptake}\right)}_{\text{with}\;\text{inhibitor}}/{\left(\text{transporter}\text{-}\text{mediated}\;\text{uptake}\right)}_{\text{without}\;\text{inhibitor}}\right).$$

For BCRP and P-gp, apparent permeability P_app_ was determined using the equation

$${\text{P}}_{\text{app}}=\left(\text{Vr}/\left(\text{A}\times \text{C}0\right)\right)\times \left(\text{Cr}/\Delta \text{t}\right)$$

whereby P_app_ is the apparent permeability, Vr is the volume of the receiver compartment (mL), C0 is the initial concentration of the donor solution, A is the monolayer area (cm^2^), Cr is the receiver well concentration at the end of the incubation, and Δt represents the change in time from t = 0 (s). The efflux ratio (ER) was determined by dividing P_app_ in the B→A direction by that in the A→B direction as:

$$\text{ER}={\text{P}}_{\text{app}}\left(\text{B}\to \text{A}\right)/{\text{P}}_{\text{app}}\left(\text{A}\to \text{B}\right).$$

Net basal (B) to apical (A) flux (B→A) of the substrate transport by BCRP and P-gp was calculated by subtracting (A→B) flux from (B→A) flux as:

$$\text{net}\;\text{B}\to \text{A}\;\text{flux}\left(\text{pmol}/\text{min}/{\text{cm}}^{2}\right)=\left(\text{B}\to \text{A}\right)\text{flux}\text{-}\left(\text{A}\to \text{B}\right)\text{flux}.$$

Percent inhibition was calculated by dividing the net flux (B→A) in the presence of the test article or the reference inhibitor by the net flux (B→A) in the absence of the inhibitor as:

$$\text{\%}\;\text{inhibition}=100-\left(100\times \text{net}\;{\text{flux}}_{\text{with}\;\text{inhibitor}}/{\left(\text{mean}\;\text{net}\;\text{flux}\right)}_{\text{without}\;\text{inhibitor}}\right).$$

For BSEP, the mean substrate accumulation in vesicles treated with AMP was subtracted from the substrate accumulation in vesicles treated with ATP as:

$$\text{vesicular}\;\text{accumulation}\left(\text{ATP}\text{-}\text{dependent}\right)\left(\text{pmol}/\text{min}/\text{mg}\right)={\left(\text{vesicular}\;\text{accumulation}\right)}_{\text{ATP}}\text{-}{\left(\text{mean}\;\text{vesicular}\;\text{accumulation}\right)}_{\text{AMP}}.$$

Signal to noise for the BSEP probe substrate was calculated by dividing the uptake of the probe substrate in BSEP-expressing vesicles in the presence of ATP by the uptake of the BSEP substrate in the vesicles in the presence of AMP. Percent inhibition in the presence of the test article or the reference inhibitors was calculated as:

$$\%\;\text{inhibition}=100-\left(\frac{100\times {\left(\text{ATP}-\text{dependent},\text{transportor}-\text{mediated}\;\text{accumulation}\right)}_{\text{with}\;\text{inhibitor}}}{{\left(\text{ATP}-\text{dependent},\text{transportor}-\text{mediated}\;\text{accumulation}\right)}_{\text{without}\;\text{inhibitor}}}\right).$$

## Author Contributions

Conceptualization, C.S.S., R.Z.Y., and Y.W.; Methodology, M.S.W., M.L., S.L., M.J., B.N., and N.P.; Investigation, M.J., B.N., N.P., and J.H.; Writing – Original Draft, C.S.S., R.Z.Y., and Y.W.; Writing – Review and Editing, C.S.S., R.Z.Y., Y.W., E.H., and D.A.N.; Funding Acquisition, S.P.H.; Resources, T.W.; Supervision, C.S.S. and R.Z.Y.
